# Co-design of a question prompt list about pregnancy and childbearing for women with polycystic kidney disease: an exploratory sequential mixed-methods study

**DOI:** 10.1186/s12884-023-06154-8

**Published:** 2023-12-11

**Authors:** Sara Holton, Craig Nelson, Bodil Rasmussen, Vicki Levidiotis

**Affiliations:** 1 School of Nursing and Midwifery, Faculty of Health, Deakin University, Geelong, VIC 3220 Australia; 2 The Centre for Quality and Patient Safety Research in the Institute of Health Transformation, Deakin University - Western Health Partnership, St Albans, VIC 3021 Australia; 3 Department of Nephrology, Western Health, St Albans, VIC 3021 Australia; 4 Department of Medicine - Western Health, University of Melbourne, Parkville, VIC 3010 Australia; 5 Western Health Chronic Disease Alliance, Western Health, St Albans, VIC 3021 Australia; 6 Department of Medicine, Deakin University, Geelong, VIC 3220 Australia; 7 Faculty of Health Sciences, University of Southern Denmark and Steno Diabetes Center, Copenhagen, Denmark; 8 Faculty of Health and Medical Sciences, University of Copenhagen, Copenhagen, Denmark

**Keywords:** Polycystic kidney disease, Pregnancy, Childbearing, Question prompt list, Australia, Co-design, Mixed-methods

## Abstract

**Background:**

Although women with polycystic kidney disease (PKD) generally have healthy pregnancies and babies, pregnancy is associated with a greater risk of maternal complications and requires planning and management of their condition. Given these possible complications, routine communication about childbearing between women with PKD and their treating team is important. A question prompt list (QPL), a structured list of questions used by patients during consultations with healthcare providers, may be beneficial in assisting women with PKD to discuss their childbearing concerns with, and seek related information from, their treating team. The aims of this study were to co‐design a QPL about pregnancy and childbearing for women with PKD, and evaluate its comprehensibility, salience, and acceptability.

**Methods:**

An exploratory sequential mixed‐methods study of women of reproductive age with PKD living in Australia, using an experience‐based co‐design approach with two phases. Women were recruited from a metropolitan public health service and via social media and invited to complete an anonymous online survey about the development of the PKD QPL (phase one) and participate in an online discussion group about its refinement (phase two).

**Results:**

Sixteen women completed the development survey and seven participated in the evaluation discussion group. Participants reported that women with PKD would value and use a QPL to prompt discussions with and seek further information about pregnancy and childbearing from their healthcare providers. Women identified four main topics for the QPL: ‘thinking about having a baby’, ‘pregnancy’, ‘my medications’ and ‘after my baby is born’. Within each section a series of questions was developed. Based on the findings, a QPL about pregnancy and childbearing for women with PKD was co-designed.

**Conclusions:**

Women with PKD often find it difficult to access information and have discussions with their health care providers about pregnancy and childbearing. The PKD QPL co-designed in this study was perceived to be an acceptable tool which will, from the perspectives of participants, assist women with PKD to access information more easily about pregnancy, childbearing and PKD; ask more targeted questions of their treating team; and make informed childbearing decisions.

**Supplementary Information:**

The online version contains supplementary material available at 10.1186/s12884-023-06154-8.

## Background

Polycystic kidney disease (PKD) is a common genetic disorder that affects up to one in 1,000 people [1–3]. There are two types of PKD: autosomal dominant polycystic kidney disease (ADPKD) and autosomal recessive polycystic kidney disease (ARPKD) with ADPKD being the most common form. There is no evidence that PKD affects fertility in women with normal renal function [1, 4]. However, pregnancy in women with PKD is associated with a higher risk of adverse maternal outcomes including hypertension, proteinuria, oedema, urinary tract infection, renal dysfunction, and preeclampsia [5]. Little is known about pregnancy outcomes for patients with ARPKD [6].

It is recommended that discussions about pregnancy and childbearing should be a routine part of clinical care for women of reproductive age with chronic kidney disease (CKD) including PKD given the increased risks and need for additional pregnancy care [1]. Yet, few women with CKD report receiving counselling about contraception or pregnancy, and many report a lack of ownership in the childbearing decision‐making process [7]. It is important that women with CKD receive information about pregnancy and childbearing so that they can make informed decisions and optimize the outcomes for themselves and their baby [7].

Nevertheless, women who have a chronic health condition such as PKD often have concerns about fertility and childbearing but find it difficult to obtain accurate, up to date information particularly about whether they can conceive; the impact of their condition and treatment (especially medications) on their baby and breastfeeding; whether their baby will inherit their condition; and the impact of pregnancy and childbirth on their future health [8–10]. Women often seek reproductive information from a range of sources including the internet, other women with the condition, and patient associations and support groups [8, 9]. However, women frequently report that they are unable to access reproductive information relevant to their needs, and there is little opportunity to discuss their fertility and childbearing concerns with their health care providers as consultations are usually focused on the treatment of their condition [8–10].

There is little evidence and guidance about how to communicate fertility and childbearing information effectively for women with CKD within a patient‐centred shared decision‐making framework [11]. Recent Australian studies have identified the need for more insights into the perspectives and preferences of women with CKD about fertility and childbearing decisions, and the development of resources such as decision aids that integrate evidence and patient preferences [11].

A question prompt list (QPL), a structured list of questions used by patients during consultations with health care providers, may assist women with PKD to discuss their childbearing concerns with, and seek related information from, their treating team. QPLs are designed to empower patients and optimise patient‐centred care and have been found to assist patients articulate their concerns, discuss difficult or sensitive matters, and ask more targeted questions [12–15]. QPLs have mainly been developed for patients with cancer [16–18] or specific chronic health conditions [12, 19], none exist about childbearing and PKD.

Without adequate information about PKD and childbearing, women may find it difficult to make informed decisions about if, when and how many children they have. The aims of this study were to co‐design a QPL about pregnancy and childbearing for women with PKD, and evaluate its comprehensibility, salience, and acceptability.

## Methods

### Study design

**Fig. 1:**
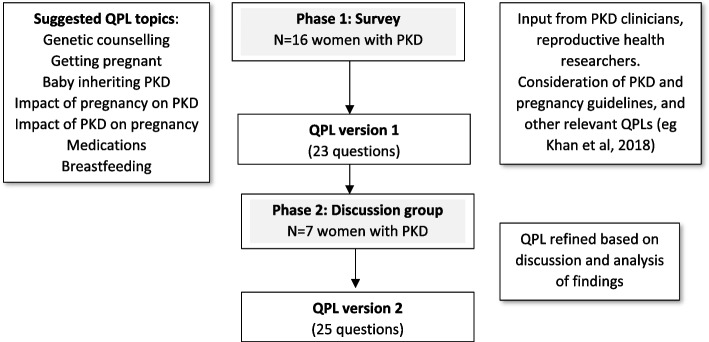
Development of a QPL about pregnancy and childbearing for women with PKD including study phases

An exploratory sequential mixed‐methods study of women of reproductive age with PKD living in Australia, using an experience‐based co‐design approach with two phases (phase 1: QPL development and phase 2: QPL refinement) (Fig. 1). The methodology was based on previous studies which have developed QPLs for people with specific health conditions [12–14, 19].

### Sample and recruitment

English‐speaking women of reproductive age (18‐45 years) with PKD living in Australia were recruited from the renal service at a public acute and sub-acute health service in metropolitan Melbourne, Australia and via advertisements on social media (Supplementary material). Women were invited to participate in phase one and/or phase two of the study. It was anticipated that an approximate sample of 15–20 women would provide sufficient information power for descriptions of different fertility concerns and information needs and contribute new knowledge [20].

### Procedure

Data were collected by a study‐specific, anonymous, self‐administered online questionnaire (phase 1: QPL development) which identified women’s information needs and preferences, and informed the development of the QPL; and an online discussion group (phase 2: QPL refinement) which revised the QPL based on the survey findings and participants’ feedback, and explored the QPL’s comprehensibility, salience and acceptability.

Women were invited to participate in the study by direct approaches from members of the research team employed at the participating health service or via advertisements on social media and newsletters of the funding organisation. A member of the research team (SH) contacted interested women to answer any questions they may have about the study and emailed the Participant Information and Consent Form which provided more details about the study and the link to the online survey (phase 1: QPL development).

#### Phase 1: QPL development

The questionnaire (phase 1: QPL development) was developed by the research team who are renal clinicians and reproductive health researchers, and based on PKD and pregnancy clinical guidelines [1], relevant literature [7, 10, 11, 21, 22], and similar QPLs for women with chronic health conditions (e.g. Khan et al. [12]). The questionnaire, available on REDCap an online survey platform, assessed respondents’ sociodemographic characteristics, likely use of a PKD QPL, and perceptions about a PKD QPL including topics to be included such as fertility, pregnancy and kidney function, medications, inheritability. Respondents were also able to provide free‐text comments (Supplementary material).

The survey was available for six weeks (8 September – 19 October 2022).

#### Phase 2: QPL refinement

An online discussion group hosted on WhatsApp refined the PKD QPL based on the findings from phase 1. At the end of the survey (phase 1), respondents were asked to identify if they would be interested in participating in an online discussion group about the refinement of the QPL (phase 2). Women who expressed interest were redirected to another survey where they provided their name and contact details (the phase 1 survey was anonymous). A member of the researcher team then contacted each woman and invited them to join the discussion group and provided the link for the WhatsApp group.

A ‘private’ group was created on WhatsApp, a free multiplatform messaging app. The group moderator (SH) sent each woman who agreed to participate a ‘welcome to the group’ message inviting them to participate in the discussion by posting their responses to the questions and comments from other group members. The moderator requested that participants ensured their WhatsApp privacy settings were consistent with what they wanted to reveal to the group prior to the commencement of the discussion.

The draft PKD QPL was shared with participants of the WhatsApp group and a discussion guide (Supplementary material) was used to prompt and initiate discussion about the PKD QPL and whether further changes were required before it could be used in a clinical setting in particular, the appropriateness of the language used in the QPL, its length, and topics; whether women with PKD would use the QPL and if it would assist them to seek information about pregnancy and childbearing; the best ways for women to access the QPL; and the best time for women to receive the QPL. The PKD QPL was revised based on the findings and then shared with the participants for final comments. The moderator read participants’ responses daily and asked clarifying questions as appropriate. Only members of the study’s WhatsApp group had access to the content posted and the identity of the group members. In order to develop a summary description of the discussion group members, anonymous demographic data was sought in a brief online survey located outside the group discussion.

The group ran for four weeks (2 – 28 November 2022).

### Data analysis

Descriptive statistics were used to summarise and describe the quantitative data (survey). The data was analysed using SPSS.

The transcript of discussion group was exported from WhatsApp and pasted into a Word document. Content analysis [23] was used to analyse the free‐text comments and transcript of the discussion group. The text was coded into themes based on pre-defined content categories (e.g. the topics outlined in the WhatsApp discussion guide). The same process was used to analyse the free-text comments and transcript of the discussion group, but the analyses were conducted separately. The analysis was conducted by the first author (SH), reviewed by the other members of the research team and interpretations discussed within the team until consensus was reached. Quotes have been included to illustrate the findings. All participant quotes have been included in Supplementary Material Table 1.

## Results

### Phase 1: QPL development (survey)

#### Survey response

Twenty-six women who met the inclusion criteria were identified from the patient list of the renal unit at the participating health service. The social media advertisements reached almost 45,000 people and resulted in 255 clicks on the link to the survey. Twenty completed surveys were submitted; four were not eligible to be included in the analysis due to the respondents not living in Australia (*n* = 3; two in the USA and one in Bahrain) or not being of childbearing age (*n* = 1; 55 years).

#### Respondent sociodemographic characteristics

Sixteen women living in Australia completed a survey. On average they were aged in their early thirties; and most were born in Australia, had a postsecondary school qualification and were partnered. Most had been diagnosed with PKD in their mid-twenties and had autosomal dominant PKD. About a third were currently on dialysis and only a few had had a kidney transplant (Table 1).

**Table 1:** Survey respondent (phase 1) and discussion group participant (phase 2) sociodemographic, PKD and reproductive characteristics

Characteristic	Survey respondents *N* = 16 n (%)^a^	Discussion group participants *N* = 3 ^b^ n (%)
Age (mean, range)	33.3 (21–41)	35 (32–37)
Aboriginal or Torres Strait Islander	5 (31.3)	0 (0)
Born in Australia	12 (75.0)	3 (100)
Has a post-secondary school qualification	15 (93.8)	3 (100)
Partnered	16 (100)	3 (100)
Has a healthcare concession card	8 (50.0)	*Not assessed*
Has private health insurance	11 (68.8)	*Not assessed*
Age when diagnosed with PKD (mean, range)	26.0 (12–35)	24.3 (21–27)
Type of PKD		
*Autosomal dominant PKD*	14 (87.5)	3 (100)
*Autosomal recessive PKD*	2 (12.5)	0 (0)
Currently on dialysis	7 (43.8)	0 (0)
Had a kidney transplant	2 (14.3)	0 (0)
Tried to get pregnant	15 (93.8)	3 (100)
Number of children (mean, range)	0.9 (0–2)	1.7 (1–2)
Desired number of children (median, range)	2 (0–4)	2 (2)
Expected number of children (median, range)	2 (0–3)	2 (2)
Taken longer than 12 months to get pregnant	4 (25.0)	0 (100)

^a^due to missing data for some items the percentages may not always equal 100

^b^the phase 2 demographic survey was only completed by three out of the seven discussion group participants

#### Reproductive experiences and desires

Most women had tried to get pregnant and the average number of the children the respondents had was about one. Most wanted to have two or more children but expected they would actually have two or less. Of those who had tried to get pregnant, almost a quarter said they had experienced fertility problems (Table 1).

#### QPL about pregnancy and childbearing

Although most women (*n* = 11, 73.3%) said they were comfortable talking to their health care providers about pregnancy and childbearing, all (*n* = 16, 100%) said they would be very likely or likely to use a QPL about pregnancy and childbearing for women with PKD.

##### Reasons for using a QPL

Women said a QPL would be helpful as it would help remind them what questions they wanted to ask or help them to ask more questions about pregnancy and childbearing when they had appointments with their healthcare providers (because sometimes they forgot what they wanted to ask or got ‘overloaded’ with information), they did not always know exactly what to ask, they did not have much knowledge about pregnancy and PKD, or they wanted to get more information about the risks and impacts of pregnancy and PKD, particularly to allow them to make informed childbearing decisions.

Most women (*n* = 13, 86.7%) thought their healthcare providers would be supportive of them using a QPL about pregnancy and childbearing in their consultations.

##### QPL topics

The women identified several concerns or matters they wanted to discuss with their health care providers and thought should be included in the PKD QPL. These included possible pregnancy risks, potential fertility difficulties, and preconception care and planning; whether their baby would inherit PKD, genetic counselling and preimplantation genetic diagnosis; the impact of pregnancy on PKD and the impact of PKD on pregnancy including how kidney function might be affected; whether women with PKD can have healthy babies; and the safety of PKD medications during pregnancy and breastfeeding (Tables 2 & 3).

**Table 2:** Pregnancy, childbearing and PKD discussions with health care providers

**Discussion topics** (‘yes’ responses)	**Topics women would like to discuss with their health care providers**	**Topics women find or think might be difficult to discuss with their health care providers**
	**n (%)**	
Need to genetic counselling and pre-implantation genetic diagnosis prior to pregnancy (*n* = 16)	8 (50.0)	3 (18.8)
Getting pregnant (*n* = 16)	7 (43.8)	4 (25.0)
Baby inheriting PKD (*n* = 16)	7 (43.8)	3 (18.8)
Impact of pregnancy on PKD (*n* = 16)	7 (43.8)	3 (18.8)
Impact of PKD on pregnancy (*n* = 16)	7 (43.8)	3 (18.8)
PKD medications – safe to use during pregnancy (*n* = 16)	5 (31.3)	1 (6.3)
PKD medications – safe to use while breastfeeding (*n* = 16)	5 (31.3)	1 (6.3)
OK to breastfeed given PKD (*n* = 16)	1 (6.3)	1 (6.3)

**Table 3:** Topics that should be included in a pregnancy and childbearing QPL for women with PKD

**Topic** (‘yes’ responses)	**N (%)**
Need for genetic counselling and pre-implantation genetic diagnosis (*n* = 16)	8 (50.0)
Risks for women with PKD getting pregnant (*n* = 16)	8 (50.0)
If women with PKD can have healthy babies (*n* = 16)	6 (37.5)
Risk of baby inheriting PKD (*n* = 16)	6 (37.5)
Impact of PKD and kidney function on pregnancy (*n* = 16)	6 (37.5)
Impact of pregnancy on PKD and kidney function (*n* = 16)	6 (37.5)
Whether PKD medications can be used during pregnancy and breastfeeding (*n* = 16)	6 (37.5)
Things should do before getting pregnant (*n* = 16)	5 (31.3)
If women with PKD can get pregnant (*n* = 16)	4 (25.0)

Most women (*n* = 11, 73.3%) thought that a QPL would be extremely or very helpful in assisting them to discuss pregnancy and childbearing with their health care provider.

##### Accessing and using a QPL

Most women said they would like to access the PKD QPL either via a website (e.g. PKD Australia or their health service) or a mobile phone app. A few said their preference was to receive the QPL in person directly from their health service or health care provider (Table 4). Most women (*n* = 14, 93.3%) felt that a QPL would assist them to ask more questions about pregnancy and childbearing and PKD in future appointments with their health care providers.

**Table 4:** Preferred ways to access PKD QPL

Access method (‘yes’ responses)	N (%)
PKD Australia website (*n* = 16)	10 (62.5)
Health service (website) (*n* = 16)	8 (50.0)
Mobile phone app (*n* = 16)	7 (43.8)
Health service (in person) (*n* = 16)	5 (31.3)
Health care provider (*n* = 16)	4 (25.0)

Women said they would use the QPL with a range of health care providers but particularly their nephrologist, obstetrician/gynaecologist, and general practitioner (GP) (Table 5). ‘Nephrologist and obstetrician/gynaecologist’ was the most common combination of health care providers reported by the survey respondents (*n* = 8, 50%) followed by ‘nephrologist, obstetrician/gynaecologist and GP’ (*n* = 6, 37.5%).

**Table 5:** Health care providers women would use PKD QPL with

Health care providers (‘yes’ responses)	N (%)
Nephrologist (*n* = 16)	12 (75.0)
Obstetrician/gynaecologist (*n* = 16)	9 (56.3)
GP (*n* = 16)	9 (56.3)
Genetic counsellor (*n* = 16)	5 (31.3)
Dietician (*n* = 16)	3 (18.8)
Pharmacist (*n* = 16)	1 (6.3)
Psychologist (*n* = 16)	1 (6.3)

Women felt the best times to receive the QPL would be before they have an appointment with their health care provider (*n* = 6, 40.0%) or at the time of PKD diagnosis (*n* = 5, 33.3%). Less preferred times were when thinking about having a baby (*n* = 2, 13.3%) or when they are discussing PKD management options with their health care provider (*n* = 1, 6.7%).

### Phase 2—QPL refinement (online discussion group)

#### Response and participant sociodemographic characteristics

Seven women participated in the online discussion group about the QPL.

Participants were invited to complete a brief demographic survey outside of the discussion group; three women (42.9%) responded. All the women who completed the demographic survey were aged in their thirties, born in Australia, married, and had completed a university degree. They all had autosomal dominant PKD and had been diagnosed with PKD in their twenties. None were currently on dialysis, and none had had a kidney transplant. All had tried to get pregnant, and they had between 1–2 children, all wanted and expected they would have two children. None had experienced any fertility problems (Table 1).

#### Refinement of the PKD QPL

##### PKD QPL refinement

Women perceived the PKD QPL to be an appropriate length, comprehensive, with clear instructions about how it should be used and agreed the questions should be categorised into separate sections for each topic (Supplementary Table 1).

*I really like it! It seems super comprehensive and definitely a lot of things I thought about before my pregnancy.‬ (Discussion group participant #1)*

*I wouldn’t want to go too much longer as 2 pages is generally where interest is kept, time is also allowed (during appointments) and information won’t be overloading. I think it is a pretty good length and not too wordy.‬ (Discussion group participant #6)*

The QPL’s format was also modified based on the participants’ feedback such as the inclusion of additional lines after individual questions to make the QPL easier to read and provide women with space to write comments and further questions.

##### PKD QPL benefits and use

The participants felt that the PKD QPL would be an ‘invaluable tool’, assist women with PKD to have discussions with their health care providers and obtain pregnancy and childbearing information, and they would use it and recommend it to others.

*I think [the QPL] will definitely help … to get conversations flowing and ensure an individual with PKD has the information needed to keep informed in their pregnancy journey. (Discussion group participant #6)*

Participants commented that they would be most likely to use the PKD QPL with their obstetrician/gynaecologist or nephrologist. Few thought they would use it with a GP as they did not believe that GPs had sufficient knowledge and expertise about PKD.

*I would definitely have used it if it was available to me prior to my pregnancies. I would have used it with both my OB and Nephrologist.‬ (Discussion group participant #6)*

*I would probably call out a nephrologist to ask questions to. As others have mentioned, most GPs don’t seem well versed in PKD.‬ (Discussion group participant #1)*

Women had different views about the best time to use the PKD QPL; some thought at the time of diagnosis whilst others felt it would not be needed until planning a pregnancy.

*Knowing the right questions to ask at diagnosis would be great. I was diagnosed at 21 and nowhere near ready for children but knowing once diagnosed that I could access a QPL to use pre pregnancy would be extremely advantageous.‬ (Discussion group participant #6)*

*I probably would have been too overwhelmed when I first got diagnosed. Maybe when family planning conversations begin with the GP and nephrologist?‬ (Discussion group participant #1)*

Women suggested a range of ways to access the PKD QPL including via a website and a printed brochure available from health care providers.

*If I’m enroute to a doctor’s appointment, having easy access via a website or something I could screen shot would be helpful … Maybe even an old school brochure to put in specialist clinics too. I haven’t had an in-person nephrology appointment for a while, but I always have a look at the selection of brochures at the GP.‬ (Discussion group participant #1)*

##### PKD QPL topics

Women identified four main topics for the PKD QPL: ‘thinking about having a baby’, ‘pregnancy’, ‘my medications’ and ‘after my baby is born’. Women also suggested improvements to each section including reordering of the questions and additional questions about the impact of pregnancy on other parts of the body, the need for additional tests (e.g. blood tests) or treatment options (e.g. dialysis) while pregnant, and which health care providers should be involved in pregnancy care for women with PKD (Supplementary Table 1).

The final version of the PKD QPL incorporated the participants’ comments and suggestions (Supplementary Material).

## Discussion

This study co-designed a QPL about pregnancy and childbearing for women with PKD, and assessed its acceptability, salience and comprehensibility. Women identified that a QPL would be beneficial in assisting them to have discussions with their health care providers, obtain information, and make informed decisions about pregnancy and childbearing. Women felt the PKD QPL should include questions about pre-pregnancy planning, pregnancy, medications, and the postnatal period; and perceived the PKD QPL to be comprehensive and relevant to their needs. Based on feedback from the participants, the PKD QPL was refined to ensure its acceptability and that it would be useful and feasible for women to use with their healthcare providers.

Women with a chronic health condition often find it difficult to access up to date relevant information about pregnancy and childbearing [8–10]. The women who participated in this study also reported that they lacked knowledge about PKD and pregnancy and childbearing and were often unable, or found it difficult, to discuss these topics with their health care providers. Therefore, QPLs can be useful tools which assist women to prompt discussions with their treating team and assist them to obtain the information they need [14].

QPLs have been shown to increase the number of questions that patients ask of their healthcare providers, improve their involvement in shared decision making and reduce their unmet information needs [14, 15, 24, 25]. The women in this study perceived that the PKD QPL would enable them to discuss pregnancy and childbearing more easily with their health care providers. Although the PKD QPL was not tested in a clinical setting, other studies have concluded that QPLs assist patients to discuss their condition and its impacts with their treating team [12, 14].

Similar to other research about QPLs for women with chronic health conditions [12, 14], many women in this study would prefer to receive the PKD QPL at diagnosis. This may reflect women’s desire to know about the implications of their condition and its treatment on other aspects of their lives at the time of diagnosis [14]. As a result, health care providers especially nephrologists could consider providing the PKD QPL to women at diagnosis to assist them to plan for pregnancy and childbearing.

### Strengths and limitations

The study was conducted in 2022 during the COVID-19 pandemic. The COVID‐19 pandemic had a considerable impact on health services and research conducted at health services. Infection control protocols at health services during the pandemic made it difficult to recruit participants particularly ‘face to face’. Nevertheless, the use of a survey and discussion group hosted on online platforms removed the need for women to meet in person to participate and prevented the spread of COVID-19.

The co-design approach used in this study ensured the PKD QPL was acceptable to women with PKD and met their needs and preferences. The mixed-method design enabled comprehensive evidence to be gathered about the need for a PKD QPL as well as the acceptability, salience and comprehensibility of the co-designed QPL. Although participants believed that the PKD QPL would be useful in discussions with their health care providers, further research is required to test the effectiveness and feasibility of the PKD QPL in a clinical setting from the perspectives of both women with PKD and health care providers.

Most women in the study had a postsecondary school qualification and lived in Australia. Consequently, the PKD QPL may be less acceptable for women with lower literacy levels or English language proficiency. Nevertheless, to ensure the readability and comprehensibility of the PKD QPL for a range of women, it was assessed for readability using the Flesch Reading Ease (score of 69.3) and the Flesch-Kincaid Grade Level (score of 6.6) tests available in Microsoft Word. These tests demonstrated the text in the QPL should be easily understood by people with a sixth or seventh grade US school education, and is consistent with the readability level recommended by healthcare agencies including the Centers for Disease Control and Prevention [26] and SA Health [27]. All participants were living in Australia at the time of the study and therefore, the PKD QPL may not be suitable for women in other settings.

Differences between women based on PKD type were not examined due to the small sample size and number of participants with ARPKD. Therefore, the findings may not reflect the needs and preferences of women with different types of PKD.

A third of the survey respondents identified as Aboriginal or Torres Strait Islander. Indigenous Australians are at greater risk of developing chronic kidney disease than non-Indigenous adults [28]. Thus, the sample is likely to be representative of people living in Australia with chronic kidney disease.

### Implications for pregnancy care policy and practice

The PKD QPL co-designed in this study provides women with an information gathering tool and discussion prompt to use with their health care providers. It is expected that the PKD QPL will enhance clinical care for women with PKD by improving patient-clinician communication and shared decision‐making about pregnancy and childbearing. Women will benefit from receiving appropriate personalised fertility‐related information so they can make informed decisions about childbearing. The PKD QPL will also assist health services to provide accessible, effective, and women‐centred pregnancy and childbearing support for the women with PKD who attend their services.

Given many women in this study expressed concern that some particularly non-specialist health care providers such as GPs may be unable to answer their questions, further resources for health care providers would be beneficial in ensuring women with PKD receive the information they require about pregnancy and childbearing.

## Conclusion

Given the possible complications during pregnancy associated with PKD, routine communication about childbearing between women with PKD and their treating team is important. This study has co-designed a resource (question prompt list) about pregnancy and childbearing for women with PKD which they can use in routine PKD and pregnancy care. The findings from this study indicate that women with PKD perceive the PKD QPL to be acceptable and comprehensive, and would use it to prompt discussions with and seek information about pregnancy and childbearing from their healthcare providers. Further research is required to test the effectiveness and feasibility of the PKD QPL in clinical settings.

### Supplementary Information

**Additional file 1: Supplementary table 1.** Discussion group themes and participant quotes

**Additional file 2. **Social media advertisement, Phase 1 survey, Phase 2 discussion guide, Phase 2 Participant quotes, PKD question prompt list

## Data Availability

The datasets used and/or analysed during the current study are available from the corresponding author on reasonable request.
