# Supplementary material for: Co-design of a question prompt list about pregnancy and childbearing for women with polycystic kidney disease: an exploratory sequential mixed-methods study
Source: BMC Pregnancy Childbirth. 2023 Dec 11;23:852. doi: 10.1186/s12884-023-06154-8 (PMC10714568; doi:10.1186/s12884-023-06154-8)
Supplement: Supplementary file 1 — Additional file 1: Supplementary table 1. Discussion group themes and participant quotes [file 12884_2023_6154_MOESM1_ESM.docx]

**Supplementary table 1**: Discussion group themes and participant quotes

| **Theme** | **Subtheme** | **Participant quote** |
| --- | --- | --- |
| Draft QPL | Perceptions | - I really like it! It seems super comprehensive & definitely a lot of things I thought about before my pregnancy.‬ (Participant #1) |
|  | Format/length (number, order of questions etc) | - I do appreciate that the questions have been broken into categories for ease of use. (Participant #6) - I think generally all really good questions, but should there be a prompt at the top as to who to ask these questions to? General GPs wouldn’t know most of these answers and general midwives wouldn’t either. ‬(Participant #4) - The only thing I would consider changing up is given the additional space below references, maybe using that for additional lines in the other questions you may wish to ask section. Of course this will change pending the number of questions added or removed. But I know when I start writing down questions I like to have one to a line to make it easier to read etc.‬ (Participant #6) - I wouldn’t want to go too much longer as 2 pages is generally where interest is kept, time is also allowed (during appointments) and information won’t be overloading. I think it is a pretty good length and not too wordy.‬ (Participant #6) - Yeah the length is good, I’d just be picking and choosing what I need anyways!‬ (Participant #1) - Order flows well and the questions appear to be in a logical manner. I like that they follow on from the preceding question.‬ (Participant #6) - Think it’s good to have separate sections for each topic area‬ (Participant #2) - There’s just a typo on the question about women with PKD having healthy babies‬ (Participant #2) |
|  | Wording | - Everything reads well for myself (Participant #6) - Looks very clear to me!‬ (Participant #1) |
|  | Instructions (how to use QPL) | - I found the instructions perfectly clear for me. I personally wouldn’t change anything.‬ (Participant #6) - Agreed the instructions are clear (Participant #1) - I also agree I also wouldn't change anything (Participant #7) |
| Use of QPL | Who most suitable for | - if I were one of the participants on dialysis or having received a transplant, I would have a greater desire to have access to the QPL myself. (Participant #6) |
|  | Benefits of QPL | - I think having the tool available will empower individuals and allow for greater conversations to follow whether planning a pregnancy or already being pregnant (Participant #6) - I think [the QPL] will definitely help as long as the health professional has a good understanding of PKD and is happy to spend the time providing support. But definitely enough to get conversations following and ensure an individual with PKD has the information needed to keep informed in their pregnancy journey 😊‬ (Participant #6) - Yes [the QPL] will help to provide all the information and support (Participant #7) - I know it can be hard to think of all the questions you need to ask, so having a tool that supports you is invaluable (Participant #6) |
|  | Which HCPs use with | - I agree with many of the responses regarding who the tool with be useful with ie, Ob, GP, genetic counsellor etc and found it interesting that some were unsure whether to seek genetic counselling as this never crossed my mind (Participant #6) - I think so many of these questions but GP won't be able to answer most of them only the professional knows everything about it (Participant #7) - I would probably call out a nephrologist to ask questions to. As others have mentioned, most GPs don’t seem well versed in PKD.‬ (Participant #1) - [The QPL] would definitely provide some inspiration for me and I would probably use some of the questions with my obstetrician or nephrologist for sure.‬ (Participant #1) - Yes I will definitely use [the QPL] and use questions with my obstetrics (Participant #7) - I would definitely have used it if it was available to me prior to my pregnancies. I would have used it with both my OB and Nephrologist.‬ (Participant #6) |
|  | Best time to use QPL | - I think if you have PKD and whatever other health concerns some of these questions should be asked when you get diagnosed or months or years before thinking about pregnancy - not while your already pregnant (Participant #4) - Knowing the right questions to ask at diagnosis would be great. I was diagnosed at 21 and no where near ready for children, but knowing once diagnosed that I could access a QPL to use pre pregnancy would be extremely advantageous.‬ (Participant #6) - I probably would have been too overwhelmed when I first got diagnosed. Maybe when family planning conversations begin with the GP + nephrologist?‬ (Participant #1) - I feel like not everyone has discussions with their healthcare provider before falling pregnant. I know we didn’t in our case as I have a strong family history of PKD so kinda knew how it would potentially impact the lives of our children if they inherited it. I also had my mum who has PKD go through a twin pregnancy with nil complications so I wasn’t too concerned of my health deteriorating whilst pregnant. So I feel like being aware of it at time of diagnosis may have sparked more discussions prior to falling pregnant for me. I can see that if you were unaware of family history, or had a more significant presentation of PKD being giving the QPL at time of diagnosis would be confronting/ may get lost in all the information, but for me I would have said that would have been a great time to receive something like this.‬ (Participant #6) |
|  | Accessing the QPL | - Just one comment on accessing a QPL - if I’m enroute to a doctors appointment, having easy access via a website or something I could screen shot would be helpful. Whenever I had obstetrician appointments I had questions written down so I didn’t forget (Participant #1) - PKD Australia would be a good place to start [to access QPL]. I think if GPs had access to it and could share it with us would be great too.‬ (Participant #6) - Maybe even an old school brochure to put in specialist clinics too. I haven’t had an in-person nephrology appointment for awhile, but I always have a look at the selection of brochures at the GP.‬ (Participant #1) |
|  | Outcomes (of using QPL for women; recommend to other women etc) | - Yes definitely [women] will [feel more confident making decisions about pregnancy and childbearing after using the QPL with their health care providers] (Participant #7) - It will make them feel more confident if the GP or Obstetrician has the right info‬ (Participant #1) - Yes definitely [women will recommend the QPL to other women with PKD]. (Participant #6) |
| HCP knowledge about PKD & childbearing |  | - with both my pregnancies I found it interesting that one Ob didn’t know much at all about PKD and often confused me with PCOS and the other was extremely informative and provided explanation for risks etc (Participant #6) - I also found in my two pregnancies that general midwives didn't know anything about PKD although I was seeing my Nephrologist very regularly throughout my first pregnancy and with my second pregnant I was going through a Foetal Medical Unit which specialise in those who are pregnant with other medical conditions or health concerns. (Participant #4‬) - Definitely [women] will [recommend the QPL to other women with PKD] it's a great help (Participant #7) - Definitely think they would recommend it to other women with PKD‬ (Participant #2) |
| Topics to be included in QPL |  | - All the suggested topics looked extremely relevant for me and my situation, and similar to the ones I asked (Participant #1) - I feel that the information needed for those with mild symptoms, not on medication would vary greatly from those who have more advanced PKD or on dialysis or have had a transplant. Perhaps having the QPL in sections that range from less advanced to more advanced PKD? (Participant #3) - in my case I have a few of the connective tissue genetics associated with PKD such a Mitral value regurgitation, flexible joins etc. I don't know how common it is for this with PKD but if it is common then perhaps a question prompt about how the other parts of the body will accommodate pregnancy when you have PKD. (Participant #3) - One more question I asked - will I need extra blood tests during my pregnancy to check my kidney function + extra BP monitoring. (Participant #1) - Also it’s not really a question, but a note that my obstetrician and nephrologist worked at the same hospital, so they were able to help manage my care together easily. So maybe something about getting doctors/ health care professionals to work together? (Participant #1) - I guess if people do opt to do genetic counselling and bub is likely to be born with PKD I guess asking questions around that may also be helpful? ‬(Participant #6) - There are all the questions to have a healthy baby and according to me there is nothing is missing in it (Participant #7) |
|  | Thinking about having a baby | - Also the question "Will raising a child effect my quality of life" I think should go in first section about thinking about having a baby (Participant #4) - I was thinking last night whilst lying in bed that maybe there should be a question in the “thinking about having a baby” section that includes who should be involved in my pregnancy care. I only got a referral to a nephrologist whilst pregnant because I asked for one with my first baby. I was only 21 when diagnosed and wasn’t linked to a nephrologist at that time because everything was normal with my tests etc. only reason I found out was because I asked to be tested knowing my mother has PKD. I could only assume that some people with similar situations to myself may not think to ask for a referral and things could be missed if PKD was to impact their health whilst pregnant.‬ (Participant #6) - I’m finding reading everyone’s comments very interesting-particularly around different experiences throughout pregnancy. My nephrologist was of the belief that PKD is a disease that can be eliminated so discussed genetic testing well before I thought about getting pregnant, but not at the first appointment. ‬( Participant #2) |
|  | Pregnancy | - Maybe something around during pregnancy, what are options if kidney function starts to decline (not just the impact). I think my nephrologist said everyone ends up with protein in their urine, but what that could mean for PKD folks.‬ (Participant #1) - I was told I’d be on dialysis from week 9 of my pregnancy due to my kidney function (fortunately this didn’t eventuate). At this point, I would have liked further information-not just about dialysis options, but an understanding of how people who had gone through pregnancy whilst on dialysis coped and how they then managed dialysis with a newborn baby. I’m not sure if that information is something a Doctor could provide? … I was really lucky that my kidneys ended up coping really well. It was just the practical stuff that I kind of wanted more info on-like-how tired will I be if I am on dialysis while pregnant, will I still feel ok working full time?‬ (Participant #2) - I don’t know if this is too specific- is there a recommendation for a certain type of dialysis which achieves better health outcomes whilst pregnant?‬ (Participant #2) |
|  | After baby is born | - I was in hospital for a few weeks with a kidney infection a couple of years ago and am constantly worried about how I’ll manage now with a young child if that happened.‬ (Participant #1) |
|  | Further resources | - My other thought is about the further reading, would Kidney Health Australia be a better resource as I often find international healthcare has different advice to Australia‬ (Participant #4) - It seems like what pregnant women need, or people in general with PKD is a group of other people who have been through the same experience and often have more experience with the disease than doctors or nephrologists. We aren't exactly trained experts but we all know the struggles and the uncertainties of having this disease and having children‬ (Participant #4) - I think the where to next style question for the what if scenarios are great. I mean it’s only when something happens that we often think, what next. I think knowing the true impact of pregnancy and complications whilst confronting are so important.‬ (Participant #6‬) |
| Pregnancy/childbearing experiences | Genetic testing/counselling | - No one ever suggested genetic testing to me, even my Neph during or before leading up to getting ready to become pregnant. (Participant #4) - I did get genetic testing prior to pregnancy for the purpose of being able to test the foetus should I wish to (which I didn't). (Participant #3) |
|  | Previous pregnancies | - Although I was very lucky and had hassle free pregnancies I was induced at 38 weeks with both because no one wanted to put the extra stress on my body - although no one knew exactly about PKD they were just focused on my blood pressure throughout (Participant #4) - I have really mild symptoms and my GP was really helpful in my pregnancies .she guided me in my whole pregnancies (Participant #7) - My obstetrician asked for the paed team to review my bub after birth as a precautionary measure. When I spoke to them about it they stated that it wasn’t necessary, I guess in that moment I was a bit unclear as to why the Ob would have suggested a review etc. all questions I should have asked at the time but didn’t. (Participant #6) |
|  | Impact of PKD on pregnancy | - Although this may not be the case for everyone I have Polycystic Liver Disease which is some what common for those with PKD, but other people may have other health issues relating to PKD. PLD is very effected by pregnancy because the hormones make the cysts on the liver grow (not the same for the kidneys) and if I known how bad it was going to get before I had children then I may have done things differently. ‬(Participant #4) |
