# Supplementary material for: Co-design of a question prompt list about pregnancy and childbearing for women with polycystic kidney disease: an exploratory sequential mixed-methods study
Source: BMC Pregnancy Childbirth. 2023 Dec 11;23:852. doi: 10.1186/s12884-023-06154-8 (PMC10714568; doi:10.1186/s12884-023-06154-8)
Supplement: Supplementary file 2 — Additional file 2. Social media advertisement, Phase 1 survey, Phase 2 discussion guide, Phase 2 Participant quotes, PKD question prompt list [file 12884_2023_6154_MOESM2_ESM.zip › 56536 Discussion Guide (PKD) Version 1 29 June 2022.docx]

**Pregnancy and childbearing for women with polycystic kidney disease: development and evaluation of a question prompt list.**

**WhatsApp group discussion**

**Discussion Guide**

*[Thank participants for volunteering; introduce self.]*

*[Go through informed consent process.]*

*[Discuss demographic survey; process for obtaining summary of results]*

*[Reminder that can withdraw at any time, and can choose not to contribute to particular topics.]*

*[Talk about discussion process:*

*Confidentiality;*

*Privacy settings on WhatsApp*

*Opportunity for all to ‘speak’ (post comments);*

*No right or wrong answers]*

“As you know, we surveyed women earlier this year about whether a question prompt list (QPL) about pregnancy and childbearing would be useful for women with PKD and what topics should be included. So based on what you told us we have developed a QPL. You should have received a copy of the draft QPL and I have also uploaded it onto this chat. The QPL is a list of questions women with PKD can take to their appointments with their healthcare providers to help them to ask questions/get information about pregnancy and childbearing.

We hope to use the findings of this discussion group to refine the QPL and determine whether further changes are required before it can be used by women in clinical settings with their healthcare providers. We hope the QPL assist women with PKD to more easily access information about childbearing and PKD, and ask more targeted questions of their PKD treating team, and assist women with PKD make informed childbearing decisions, achieve their reproductive goals, and reduce adverse maternal and perinatal outcomes.”

**What do you think of the QPL overall?**

**Do you think the QPL needs any changes?**

*PROMPTS (after any general discussion)*

Which sections/questions? Why/Why not?

Does it cover the relevant areas/topics (eg getting pregnant, medications, breastfeeding etc)? Should any other topics be included? Should any topics be excluded?

Is it easy to understand? Is the language used OK?

Is it the right length? Include the right number of questions?

Are the instructions about how to use it clear? Is it user-friendly?

**Do you think the QPL will help women with PKD get the information they need about pregnancy and childbearing?**

Why/Why not?

Do you think it will encourage them/make it easier for women to talk to their healthcare providers about pregnancy and childbearing?

Do you think it will help them to get the information they need to make pregnancy/childbearing related decisions?

E.g. information and decisions about whether it would be beneficial for them to have genetic counselling and pre-implantation genetic diagnosis before getting pregnant etc

**Do you think women with PKD will use the QPL?**

Why/Why not?

Do you think they will feel more confident making decisions about pregnancy and childbearing after using the QPL?

Do you think women will recommend the QPL to other women with PKD?

Would you use it? Why/Why not? Which health care providers would you use it with?

**What do you think are the best ways for women with PKD to access the QPL?**

Eg via PKD website? App? Health service? Health care provider?

**When do you think is the best time for women with PKD to be given the QPL?**

Eg when first diagnosed, when thinking about having children, when they make an appointment to see their healthcare provider, at a consultation with a healthcare provider?

**Is there anything else you would like to say about the QPL for women with PKD?**

*Thank for participation.*

*We will send results if they leave address: email preferred.*

*Request preferred contact details for gift voucher – email to CI.*

*Remind about demographic survey*
