# Supplementary material for: Co-design of a question prompt list about pregnancy and childbearing for women with polycystic kidney disease: an exploratory sequential mixed-methods study
Source: BMC Pregnancy Childbirth. 2023 Dec 11;23:852. doi: 10.1186/s12884-023-06154-8 (PMC10714568; doi:10.1186/s12884-023-06154-8)
Supplement: Supplementary file 2 — Additional file 2. Social media advertisement, Phase 1 survey, Phase 2 discussion guide, Phase 2 Participant quotes, PKD question prompt list [file 12884_2023_6154_MOESM2_ESM.zip › 56536 PKD Survey v1 21Jul22.pdf]

Developing a question prompt list about pregnancy and childbearing for women with PKD

Development and evaluation of a question prompt list about pregnancy and childbearing for women with polycystic kidney disease

Survey
Thank you for your interest in our study. This project has been approved by the Western Health Low Risk Ethics Panel.
The participant information sheet tells you more about the study and can be accessed via this link: [INSERT LINK TO PICF]
Please read through the participant information sheet.
We will not ask you to write your name on this survey.
There are no right or wrong answers to the questions in this survey. We are simply interested in your experiences, thoughts and opinions. If you are unsure about how to answer a question, please mark the response which corresponds most closely to how you feel.
The survey will take approximately 15 minutes to complete.
Your completion of the survey indicates your consent to participate in the study.
Thank you for your participation. If you have any questions about the study please contact Dr Sara Holton at email: sara.holton@wh.org.au

Section 1. Some questions about you

What was your age at your last birthday?

____
(Please enter your age in years)

Are you Aboriginal or Torres Strait Islander?

◯ Yes
◯ No

In which country were you born?

◯ Australia
◯ Other (please specify)

Please specify the country in which you were born

____

What is the highest level of education you have completed so far?	◯ Partially completed secondary school (less than Year 12)<br>◯ Completed secondary school (Year 12)<br>◯ Trade/apprenticeship (eg hairdresser, chef)<br>◯ Certificate/diploma (eg child care, technician)<br>◯ University degree<br>◯ Higher university degree (eg Master's, PhD)
Which of these best describes your current relationship status?	◯ Married (in a registered marriage)<br>◯ Living with a partner (opposite sex) in a relationship<br>◯ Living with a partner (same sex) in a relationship<br>◯ I have a boyfriend/partner (opposite sex) I don't live with<br>◯ I have a girlfriend/partner (same sex) I don't live with<br>◯ Not currently in a relationship
What is the postcode where you live?	__________________________________
Do you have a healthcare concession card?	◯ Yes<br>◯ No
Do you have private health insurance?	◯ Yes<br>◯ No
How old were you when you were diagnosed with polycystic kidney disease (PKD)?	__________________________________<br>(Please enter your age in years)
Which type of PKD do you have?	◯ Autosomal dominant PKD<br>◯ Autosomal recessive PKD
Are you on dialysis?	◯ Yes<br>◯ No
Have you had a kidney transplant?	◯ Yes<br>◯ No
Have you ever tried to get pregnant?	◯ Yes<br>◯ No
How many children do you have?	◯ 0<br>◯ 1<br>◯ 2<br>◯ 3<br>◯ 4<br>◯ 5<br>◯ 6 or more
If you could have exactly the number of children you want, what would that number be?	◯ 0<br>◯ 1<br>◯ 2<br>◯ 3<br>◯ 4<br>◯ 5<br>◯ 6 or more

Realistically, how many children do you think you will have in total (including any children you already have)?	◯ 0<br>◯ 1<br>◯ 2<br>◯ 3<br>◯ 4<br>◯ 5<br>◯ 6 or more
Has it ever taken you longer than 12 months to get pregnant?	◯ Yes<br>◯ No<br>◯ Not applicable - I have never tried to get pregnant

Section 2. Your thoughts about a question prompt list (QPL) about pregnancy and childbearing for women with PKD

A question prompt list (QPL) is a structured list of questions that can be used by patients during consultations with their health care providers to ask questions about their medical condition or health issue. We are planning to develop a QPL about pregnancy and childbearing for women who have PKD to assist them discuss their childbearing concerns with, seek related information from, and ask more targeted questions of their treating team. We hope that a QPL might help women with PKD make decisions about if, when and how many children they have.

Do you think you would use a question prompt list (QPL) about pregnancy and childbearing for women with PKD?	◯ Very likely<br>◯ Likely<br>◯ Unlikely<br>◯ Not at all
Why would you use a question prompt list (QPL) about pregnancy and childbearing?	______________________________
Why wouldn't you use a question prompt list (QPL) about pregnancy and childbearing?	______________________________
Do you think think your health care providers (eg PKD treating team) would be supportive of you using a question prompt list (QPL) about pregnancy and childbearing in your consultations with them?	◯ Yes<br>◯ No<br>◯ I don't know/I'm not sure
How comfortable are you speaking to your health care providers about pregnancy and childbearing?	◯ Very comfortable<br>◯ Comfortable<br>◯ Neutral<br>◯ Uncomfortable<br>◯ Very uncomfortable
Which aspects of pregnancy and childbearing and PKD would you like to speak with your health care providers about?	☐ Getting pregnant<br>☐ My baby inheriting PKD<br>☐ If I should have genetic counselling and pre-implantation genetic diagnosis before getting pregnant<br>☐ Which PKD medications are safe to use during pregnancy<br>☐ Which PKD medications are safe to use when I am breastfeeding my baby<br>☐ The impact pregnancy might have on my PKD<br>☐ The impact PKD might have on pregnancy<br>☐ If it will be OK to breastfeed my baby given my PKD<br>☐ Other (please specify)<br>(Select all that apply)
What other aspects of pregnancy and childbearing would you like to discuss with your health care providers?	______________________________

Which aspects of pregnancy and childbearing and PKD do you find, or think it might be, difficult to speak with your health care providers about?	☐ Getting pregnant<br>☐ My baby inheriting PKD<br>☐ If I should have genetic counselling and pre-implantation genetic diagnosis before getting pregnant<br>☐ Which PKD medications are safe to use during pregnancy<br>☐ Which PKD medications are safe to use when I am breastfeeding my baby<br>☐ The impact pregnancy might have on my PKD<br>☐ The impact PKD might have on pregnancy<br>☐ If it will be OK to breastfeed my baby given my PKD<br>☐ Other (please specify)<br>(Select all that apply)
What other aspects of pregnancy and childbearing do you find difficult to discuss with your health care providers?	__________________________________
How helpful do you think a question prompt list (QPL) would be to assist you to speak with your health care providers about pregnancy and childbearing?	◯ Extremely helpful<br>◯ Very helpful<br>◯ Somewhat helpful<br>◯ Slightly helpful<br>◯ Not at all helpful
What topics do you think should be included in a question prompt list (QPL) about pregnancy and childbearing for women with PKD?	☐ if women with PKD can get pregnant<br>☐ if there are any risks for women with PKD in getting pregnant<br>☐ if there is anything women with PKD should do before they get pregnant<br>☐ if women with PKD can have healthy babies<br>☐ the risk of their baby inheriting PKD<br>☐ if women with PKD should have genetic counselling and pre-implantation genetic diagnosis before getting pregnant<br>☐ how PKD and their kidney function may affect pregnancy<br>☐ how pregnancy may affect PKD and their kidney function<br>☐ whether PKD medications can be used during pregnancy and breastfeeding<br>☐ other (please specify)<br>(Please select all that apply)
What other aspects of pregnancy and childbearing do you think should be included in a question prompt list (QPL) for women with PKD?	__________________________________
What do you think would be the best ways for women with PKD to access the question prompt list (QPL)?	☐ The PKD Australia website<br>☐ A mobile phone app<br>☐ Their health service (in person)<br>☐ Their health service (website)<br>☐ Their health care provider<br>☐ Other (please specify)<br>(Please select all that apply)
What other ways do you think women should be able to access the question prompt list (QPL)?	__________________________________

Which health care providers would you use a question prompt list (QPL) about pregnancy and childbearing with?	☐ Nephrologist (kidney specialist)<br>☐ Obstetrician/gynaecologist<br>☐ GP<br>☐ Pharmacist<br>☐ Dietician<br>☐ Genetic counsellor<br>☐ Psychologist<br>☐ Other (please specify)<br>(Please select all that apply)
Which other health care providers would you use a question prompt list (QPL) with?	__________________________________
Do you think a question prompt list (QPL) would help you to ask more questions about pregnancy and childbearing and PKD in future appointments with your healthcare providers?	○ Yes<br>○ No<br>○ I don't know/I'm not sure
When do you think would be the best time for women to receive the question prompt list (QPL)?	○ At the time of PKD diagnosis<br>○ before they have an appointment with their health care provider<br>○ When they have an appointment with their health care provider<br>○ when they are discussing PKD management options with their health care provider<br>○ When they are thinking about having a baby<br>○ Other (please specify)<br>(Please select all that apply)
When do you think would be the best time for women with PKD to receive a question prompt list (QPL) about pregnancy and childbearing?	__________________________________

Section 3: Anything else?

Have we missed anything? If you have anything else you would like to tell us about a question prompt list (QPL) about pregnancy and childbearing for women with PKD, please write in this box.

____

Everyone who completes a survey is eligble to go in the draw for one of three $50 gift vouchers. The draw will occur in late 2022. We will email the lucky recipient.

Do you wish to enter the gift voucher draw?

◯ Yes
◯ No

Would you like to receive a summary of the findings from the project once they are available?

◯ Yes
◯ No

Once we have created the question prompt list (QPL) we would be interested to hear your thoughts about it and whether you think any changes are needed.

Would you like to participate in an online discussion group to talk about the draft QPL (everyone who participates in the discussion group will receive a $30 gift voucher)?

◯ Yes
◯ No

So that your responses to the survey remain anonymous you will be directed to a separate form to register your interest in the gift voucher draw.

Please tick the box below if you would like to go in the draw or receive a summary of the findings or participate in the online discussion group about the draft QPL.

☐ I would like to go in the gift voucher draw and/or receive a summary of the project findings and/or participate in the online discussion group.

Contact List

1) Your name

____

2) Your email address

____

3) Would you like to go in the draw for the gift vouchers?

◯ Yes
◯ No

4) Would you like to receive a summary of the results when they are available (expected to be early-mid 2023)?

◯ Yes
◯ No

5) Would you like to participate in the online discussion group which will refine the QPL?

◯ Yes
◯ No

Thank you for completing this questionnaire.

Your responses will contribute to developing a QPL about pregnancy and childbearing for women with PKD.
