# Supplementary material for: Co-design of a question prompt list about pregnancy and childbearing for women with polycystic kidney disease: an exploratory sequential mixed-methods study
Source: BMC Pregnancy Childbirth. 2023 Dec 11;23:852. doi: 10.1186/s12884-023-06154-8 (PMC10714568; doi:10.1186/s12884-023-06154-8)
Supplement: Supplementary file 2 — Additional file 2. Social media advertisement, Phase 1 survey, Phase 2 discussion guide, Phase 2 Participant quotes, PKD question prompt list [file 12884_2023_6154_MOESM2_ESM.zip › COREQ checklist 2Nov23.docx]

**Manuscript:** Co-design of a question prompt list about pregnancy and childbearing for women with polycystic kidney disease: an exploratory sequential mixed-methods study

**Consolidated criteria for reporting qualitative studies (COREQ): 32-item checklist**

| **Domain and number of items** | **Guide questions/description** | **Reported on page:** |
| --- | --- | --- |
| **Domain 1: Research team and reﬂexivity** | | |
| ***Personal Characteristics*** | | |
| 1. Interviewer/facilitator | Which author/s conducted the interview or focus group? | Page 5 |
| 2. Credentials | What were the researcher’s credentials? E.g. PhD, MD | PhD |
| 3. Occupation | What was their occupation at the time of the study? | Senior Research Fellow |
| 4. Gender | Was the researcher male or female? | Female |
| 5. Experience and training | What experience or training did the researcher have? | Page 6 |
| ***Relationship with participants*** | | |
| 6. Relationship established | Was a relationship established prior to study commencement? | Page 5 |
| 7. Participant knowledge of the interviewer | What did the participants know about the researcher? e.g. personal goals, reasons for doing the research | Page 5 The researchers contacted women prior to the commencement of the study and the participant information and consent form explained the reasons for undertaking the research. |
| 8. Interviewer characteristics | What characteristics were reported about the inter viewer/facilitator? e.g. Bias, assumptions, reasons and interests in the research topic | Page 5 Details about the facilitator were included in the participant Information and consent form, and the facilitator introduced herself to the group at the commencement of the online discussion group. |
| **Domain 2: study design** | | |
| ***Theoretical framework*** | | |
| 9. Methodological orientation and Theory | What methodological orientation was stated to underpin the study? e.g. grounded theory, discourse analysis, ethnography, phenomenology, content analysis | Page 4 |
| ***Participant selection*** | | |
| 10. Sampling | How were participants selected? e.g. purposive, convenience, consecutive, snowball | Page 5 |
| 11. Method of approach | How were participants approached? e.g. face-to-face, telephone, mail, email | Page 5 |
| 12. Sample size | How many participants were in the study? | Page 7 & 9 |
| 13. Non-participation | How many people refused to participate or dropped out? Reasons? | Page 7 |
| ***Setting*** | | |
| 14. Setting of data collection | Where was the data collected? e.g. home, clinic, workplace | Page 5 -6  . |
| 15. Presence of non-participants | Was anyone else present besides the participants and researchers? | N/A |
| 16. Description of sample | What are the important characteristics of the sample? e.g. demographic data, date | Table 1 |
| ***Data collection*** | | |
| 17. Interview guide | Were questions, prompts, guides provided by the authors? Was it pilot tested? | Supplementary material |
| 18. Repeat interviews | Were repeat inter views carried out? If yes, how many? | N/A |
| 19. Audio/visual recording | Did the research use audio or visual recording to collect the data? | N/A The online discussion group was hosted on WhatsApp and the transcript exported from WhatsApp (p5-6) |
| 20. Field notes | Were ﬁeld notes made during and/or after the interview or focus group? | N/A |
| 21. Duration | What was the duration of the interviews or focus group? | Page 6 |
| 22. Data saturation | Was data saturation discussed? | Page 5, it was anticipated that a sample of 15-20 women would provide sufficient information power. |
| 23. Transcripts returned | Were transcripts returned to participants for comment and/or correction? | N/A, the online discussion group participants had access to the transcript via WhatsApp. |
| **Domain 3: analysis and ﬁndings** | | |
| ***Data analysis*** | | |
| 24. Number of data coders | How many data coders coded the data? | Page 7 |
| 25. Description of the coding tree | Did authors provide a description of the coding tree? | The themes are included in Supplementary Material table 1. |
| 26. Derivation of themes | Were themes identiﬁed in advance or derived from the data? | Page 7 |
| 27. Software | What software, if applicable, was used to manage the data? | Page 7 |
| 28. Participant checking | Did participants provide feedback on the ﬁndings? | Page 6 |
| ***Reporting*** | | |
| 29. Quotations presented | Were participant quotations presented to illustrate the themes/ﬁndings? Was each quotation identiﬁed? e.g. participant number | Illustrative quotes are included in the Results section and all quotes are included in Supplementary Material Table 1. |
| 30. Data and ﬁndings consistent | Was there consistency between the data presented and the ﬁndings? | Pages 7-12 |
| 31. Clarity of major themes | Were major themes clearly presented in the ﬁndings? | Pages 10-12 |
| 32. Clarity of minor themes | Is there a description of diverse cases or discussion of minor themes? | Pages 10-12 |

**Reference**:

Tong, A., Sainsbury, P., & Craig, J. (2007) Consolidated criteria for reporting qualitative research (COREQ): a 32-item checklist for interviews and focus groups. *International Journal for Quality in Health Care*, *19*(6), 349 – 357. <https://doi.org/10.1093/intqhc/mzm042>.
