# Supplementary material for: Co-design of a question prompt list about pregnancy and childbearing for women with polycystic kidney disease: an exploratory sequential mixed-methods study
Source: BMC Pregnancy Childbirth. 2023 Dec 11;23:852. doi: 10.1186/s12884-023-06154-8 (PMC10714568; doi:10.1186/s12884-023-06154-8)
Supplement: Supplementary file 2 — Additional file 2. Social media advertisement, Phase 1 survey, Phase 2 discussion guide, Phase 2 Participant quotes, PKD question prompt list [file 12884_2023_6154_MOESM2_ESM.zip › PKD demographic survey (WhatsApp group).pdf]

Default Question Block

Pregnancy and childbearing QPL for women with polycystic kidney disease

Demographic survey

Thank you once again for agreeing to be part of our discussion group.
So that we can summarise the characteristics of the people who participated in the group, we would be grateful if you could please answer the following questions.

How old are you?

Are you a Aboriginal or Torres Strait Islander?

- Yes

- [ ] No

In which country were you born?

[ ]

What is the highest level of education you have completed so far?

- [ ] Partially completed secondary school (less than Year 12)
- [ ] Completed secondary school (Year 12)
- [ ] Trade/apprenticeship (eg hairdresser, chef)
- [ ] Certificate/diploma (eg child care, technician)
- [ ] University degree
- [ ] Higher university degree (eg Master's, PhD)

Which of these best describes your current relationship status?

- [ ] Married (in a registered marriage)
- [ ] Living with a partner (opposite sex) in a relationship
- [ ] Living with a partner (same sex) in a relationship
- [ ] I have a boyfriend/partner (opposite sex) I don't live with
- [ ] I have a girlfriend/partner (same sex) I don't live with
- [ ] Not currently in a relationship

What is the postcode where you live?

[ ]

How old were you when you were diagnosed with polycystic kidney disease (PKD)?

[ ]

Which type of PKD do you have?

- [ ] Autosomal dominant PKD
- [ ] Autosomal recessive PKD

Are you on dialysis?

- Yes
- No

Have you had a kidney transplant?

- Yes
- No

Have you ever tried to get pregnant?

- Yes
- No

How many children do you have?

____

If you could have exactly the number of children you want, what would that number be?

____

Realistically, how many children do you think you will have in total (including any children you already have)?

____

Has it ever taken you longer than 12 months to get pregnant?

- Yes
- No
- Not applicable – I have never tried to get pregnant

Thank you for completing this survey!

Deakin University CRICOS Provider Code 00113B.

Powered by Qualtrics
