# Supplementary material for: Co-design of a question prompt list about pregnancy and childbearing for women with polycystic kidney disease: an exploratory sequential mixed-methods study
Source: BMC Pregnancy Childbirth. 2023 Dec 11;23:852. doi: 10.1186/s12884-023-06154-8 (PMC10714568; doi:10.1186/s12884-023-06154-8)
Supplement: Supplementary file 2 — Additional file 2. Social media advertisement, Phase 1 survey, Phase 2 discussion guide, Phase 2 Participant quotes, PKD question prompt list [file 12884_2023_6154_MOESM2_ESM.zip › PKD QPL 1Mar23.docx]

**Question prompt list about pregnancy and childbearing for women with polycystic kidney disease**

This question prompt list (QPL) is for women who have polycystic kidney disease (PKD). The QPL includes questions you may want to ask your doctor or other health care provider about pregnancy and childbearing.

You don’t have to ask all the questions or ask them exactly the way they are written. You can adapt or change them depending on your circumstances; different questions may be important to you at different times.

***Questions you might want to ask your doctor or other health care provider:***

| **Thinking about having a baby** |
| --- |

- Will my baby inherit PKD?
- Should I have genetic counselling before getting pregnant (i.e. to discuss whether my baby might get the gene for PKD and what my options are)?
- Should I have preimplantation genetic diagnosis (i.e. testing an embryo after IVF for particular genetic conditions)?
- Will having PKD make it difficult for me to get pregnant?
- Is there anything I should do before I try to get pregnant?
- Is there anything I should do to increase my chances of getting pregnant?
- Will I need IVF or fertility treatment to get pregnant?
- Are there any risks for me in getting pregnant due to my PKD?
- Can women with PKD get pregnant?
- Can women with PKD can have healthy babies?
- Will any other health conditions I have affect my ability to get pregnant/have a healthy pregnancy/baby?
- Where can I get more information about PKD and pregnancy?

| **Pregnancy** |
| --- |

- What impact will pregnancy have on my PKD?
- What impact will PKD have on my pregnancy?
- Will my kidney function have any effect on my pregnancy?
- Will pregnancy affect my kidney function?
- Will I need to/can I have dialysis while I am pregnant? Will dialysis while I am pregnant negatively affect my energy levels?
- Will I need any special/additional health care in pregnancy because of my PKD?
- How will my PKD be managed while I am pregnant?
- Will I feel well enough when I am pregnant to work (paid employment) given my PKD?

| **My medications** |
| --- |

- Which PKD medications are safe to use during pregnancy?
- Which PKD medications are safe to use during breastfeeding

| **After my baby is born** |
| --- |

- If OK to breastfeed my baby given my PKD?
- Will raising a child affect my quality of life?
- What is the best way to manage my PKD (including dialysis etc) and care for a baby?

| **Your questions** |
| --- |

You may have other questions you would like to discuss with your doctor or health care provider. You can write them here, this may help you to remember what you would like to ask at your next appointment.

**________________________________________________________________________________________________________________________________________________________________________________________________________________________________________________________________________________________________________________________________________________**

**____________________________________________________________________________________**

**____________________________________________________________________________________**

**Other resources about PKD and pregnancy and childbearing**

You may also find these resources useful:

- PKD Foundation (UK) – Pregnancy and PKD: [Pregnancy and PKD | PKD Foundation (pkdcure.org)](https://pkdcure.org/living-with-pkd/pregnancy-and-pkd/#:~:text=It%20is%20important%20for%20a,prevented%20from%20getting%20enough%20blood.)
- PKD Charity (UK) - ADPKD and pregnancy: [ADPKD and pregnancy (pkdcharity.org.uk)](https://pkdcharity.org.uk/adpkd/life-with-adpkd/adpkd-and-pregnancy)
