# Supplementary material for: Co-design of a question prompt list about pregnancy and childbearing for women with polycystic kidney disease: an exploratory sequential mixed-methods study
Source: BMC Pregnancy Childbirth. 2023 Dec 11;23:852. doi: 10.1186/s12884-023-06154-8 (PMC10714568; doi:10.1186/s12884-023-06154-8)
Supplement: Supplementary file 2 — Additional file 2. Social media advertisement, Phase 1 survey, Phase 2 discussion guide, Phase 2 Participant quotes, PKD question prompt list [file 12884_2023_6154_MOESM2_ESM.zip › Social media ad (Sep 22).docx]

**Social media- advertisement**


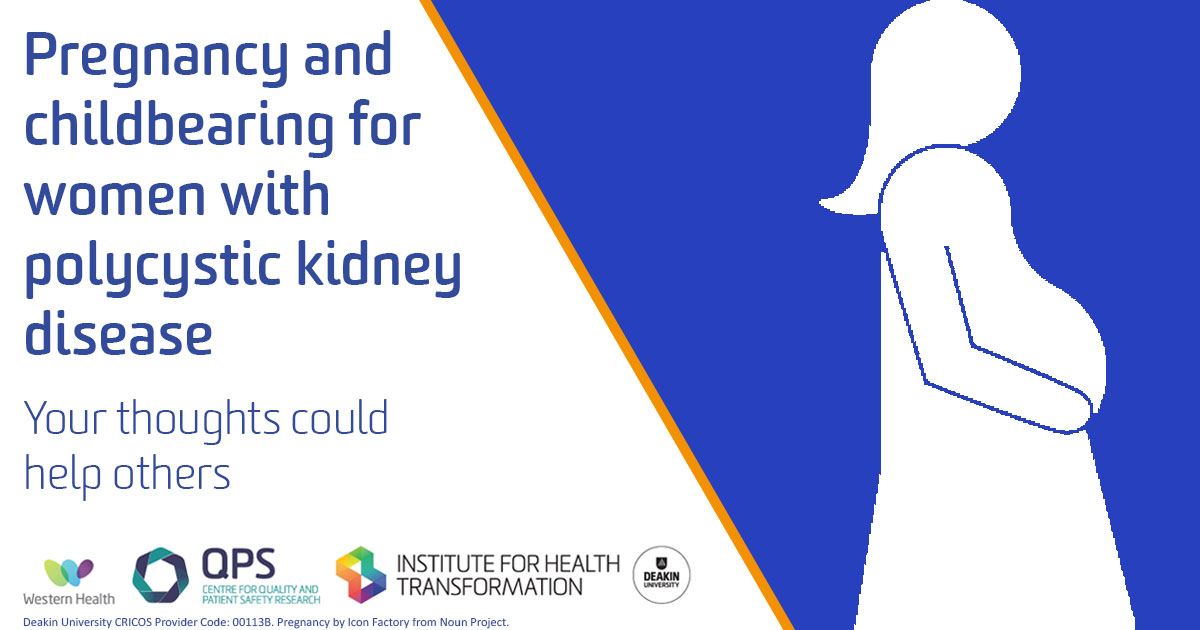


Copy: We are looking for women with polycystic kidney disease aged 18 years or more to complete a brief online survey and/or participate in an online discussion group. The research will develop a question prompt list for women with PKD which they can use to make informed decisions about if, when and how many children they have. You will be asked to complete a brief (15 minutes) online survey and participate in an online discussion group hosted on WhatsApp. All women who participate will receive a $30 gift voucher.
